# Exploring Injury Prevention Strategies for Futsal Players: A Systematic Review

**DOI:** 10.3390/healthcare12141387

**Published:** 2024-07-11

**Authors:** João P. Oliveira, Tatiana Sampaio, Daniel A. Marinho, Tiago M. Barbosa, Jorge E. Morais

**Affiliations:** 1 Department of Sport Sciences, University of Beira Interior, 6201-001 Covilhã, Portugal; tatiana_sampaio30@hotmail.com (T.S.); dmarinho@ubi.pt (D.A.M.); 2 Research Center in Sports, Health and Human Development (CIDESD), 6201-001 Covilhã, Portugal; 3 Department of Sport Sciences, Instituto Politécnico de Bragança, 5300-253 Bragança, Portugal; barbosa@ipb.pt (T.M.B.); morais.jorgestrela@ipb.pt (J.E.M.); 4 Research Center for Active Living and Wellbeing (LiveWell), Instituto Politécnico de Bragança, 5300-253 Bragança, Portugal

**Keywords:** futsal, injury prevention programs, warm-up, multicomponent, strength

## Abstract

Futsal carries a high risk of injury for players. This systematic review aimed to assess the existing literature on injury prevention strategies for futsal players. The literature was searched using PubMed, Web of Science, and Scopus databases from inception to 20 March 2024. Relevant articles were searched using the terms “futsal” AND “injury” AND “prevention”. Fourteen studies were included in the review. The review identified several injury prevention strategies with potential benefits for futsal players. Structured warm-up routines were shown to improve balance and eccentric strength and to reduce total, acute, and lower limb injuries. Proprioceptive training methods were suggested to improve joint stability and landing mechanics, which may reduce the risk of injury. Furthermore, multicomponent methods that include components such as core stability and flexibility have shown potential for reducing injury rates in futsal players. Finally, by reducing fatigue and improving movement control, strength training procedures designed to correct muscular imbalances may improve performance, which may ultimately minimize the risk of injury. This systematic review demonstrates the potential benefits of different injury prevention strategies for futsal players. The combination of several strategies, such as proprioceptive training, multicomponent programs, warm-up routines, and strength training specifically designed to address muscular imbalances, appears promising.

## 1. Introduction

Futsal, a fast-paced and dynamic variant of football played indoors on a hard surface, has become very popular worldwide [1]. Its unique characteristics [2,3], including smaller playing areas, an emphasis on tight ball control, frequent rapid changes of direction, and the potential for collisions on a hard surface contribute to a physically demanding sport that exposes players to a high risk of injury [4]. Studies have reported higher injury rates in futsal comparable to outdoor football, with ankle sprains, muscle strains, and knee injuries being particularly common [5,6].

These injuries have a significant negative impact, not only on individual players, but also on team success and long-term health [7]. When a player is sidelined due to injury, his performance is obviously compromised, potentially creating gaps in the team’s strategy and overall success [8]. This can be particularly detrimental in a fast-paced sport such as futsal, where individual skill and coordinated team movement are paramount [9]. Furthermore, recurrent injuries can lead to long-term health problems for players, potentially forcing them into early retirement or reducing their quality of life even after their playing days are over [10].

Several studies have been conducted on the prevalence of injuries in futsal [11,12,13]. For example, Junge and Dvorak examined player injuries over three consecutive World Cups [14]. Their study used a well-established injury reporting system in which team physicians reported all injuries on standardized forms after each match. The 93% response rate verified the completeness of the data [14]. The study found an alarming injury rate, with 165 injuries reported from only 127 matches. This equates to an injury rate of 195.6 per 1000 player hours, or 130.4 per 1000 matches. Notably, the majority of injuries (70%) occurred in the lower extremities, with contact with another player being the most common cause. The most common diagnoses were lower-leg contusion (11%), ankle sprain (10%), and groin strain (8%) [14].

Although injury prevention strategies have been studied in football [15], the literature lacks an in-depth analysis of futsal. This is concerning given the high injury rates recorded in futsal and its unique playing environment when compared to outdoor football. Systematic research focused solely on futsal injury prevention strategies is essential to determine the most effective methods to protect players and coaches.

Effective injury prevention methods are critical for players of all skill levels. By implementing specific training programs, futsal players can reduce their risk of injury and maintain peak performance throughout the season [16,17]. Consequently, prioritizing injury prevention through well-designed training programs and proper recovery techniques is essential to optimize team performance, protect players’ physical well-being, and ensure the long-term sustainability of their futsal careers [18].

Therefore, the aim of this systematic review was to assess the existing literature on injury prevention strategies in futsal players. This will allow the identification of injury prevention programs (e.g., warm-up routines, strength training programs) and intrinsic factors related to injury that have been described in the literature. It will also allow the establishment of some recommendations for coaches and physical fitness trainers that could help to reduce the overall number of injuries in futsal players.

## 2. Materials and Methods

### 2.1. Literature Search and Article Selection

This systematic review was carried out according to the recommendations and criteria of the Preferred Reporting Items for Systematic Reviews and Meta-Analysis Statement (PRISMA) [19]. The study protocol was registered in PROSPERO under the code CRD42024526674.

A systematic review of studies identified in the Web of Science, PubMed, and Scopus databases was performed up to 20 March 2024. Relevant articles were searched using the terms “futsal” AND “injury” AND “prevention”. The search and selection of studies were performed by two independent reviewers (JO and TS). Disagreements between the two reviewers were resolved by discussion; when necessary, a third reviewer (JM) was consulted to reach a consensus.

The keywords used for the review were “futsal” and “injury” and “prevention”. Two authors (JO and TS) independently reviewed the titles and abstracts of the identified articles. Disagreements between the two reviewers were resolved by discussion; when necessary, a third reviewer (JM) was consulted to reach a consensus.

Two independent authors (JO and TS) screened the articles for inclusion and assessed eligibility. The same two authors (JO and TS) independently assessed each article in two stages of sorting: the title and abstract and then the full text of the article.

### 2.2. Inclusion and Exclusion Criteria

The eligibility criteria were structured according to the PI(E)COS framework (P: population, I(E): intervention or independent variable (or exposure), C: comparison, control, or comparator, O: outcomes, and S: study design). The population (P) studied consisted of futsal players of any age or gender. The intervention (I) included studies that used injury prevention training programs or prevention protocols as an intervention method to prevent injuries in futsal players. Training programs could include any type of training, such as strength training, proprioceptive training, and multicomponent training: balance; core stability; and functional strength and mobility. Comparators (C) were defined as studies with a control group for comparison. Therefore, the outcomes (O) analyzed were those that in some way assessed the prevalence of injury in futsal players before and after the exercise intervention program. Finally, the included studies (S) ranged from different types of studies such as cross-sectional, longitudinal, experimental, exploratory, descriptive, and randomized control trials and crossover. Summaries of lectures and review articles were excluded.

Exclusion criteria included (1) protocol studies (i.e., those that only provided a detailed description of the study hypothesis, rationale, and methodology of the study, but not the study results) and (2) grey literature, websites, and Google Scholar were not included.

### 2.3. Data Extraction

Two authors (JO and TS) independently extracted the characteristics and results of the interventions in each included publication, according to the PRISMA statement [19]. From each study, the first author, year of publication, information on the characteristics of the participants (sample size, sex of the participant, mean age, and standard deviation), duration of the intervention, frequency of the training program, the instrument used to assess the injury, and the main outcomes were extracted.

### 2.4. Risk of Bias and Quality Evaluation of Study’s Quality

The Downs and Black Quality Assessment Checklist was used to assess the quality of each article [20]. The original version has 27 items with a maximum score of 32 points. Adjustments were made to the original version according to the focus of the included studies and the previously modified versions. For example, items 4, 8, 9, 14, 15, 17, 19, and 22 to 26 were excluded if not applicable to the study design (i.e., cross-sectional study), and the last item was configured as “yes” (1 point) or “no” (0 points), instead of five points as described by others [21,22], resulting in a maximum score of 17 points. Quality was classified as follows: (i) low, if the score was ≤50%; (ii) good, if the score was between 51% and 75%; and (iii) excellent, if the score was >75% [23]. Agreement between two independent reviewers was calculated using Cohen’s Kappa coefficient and interpreted as follows: (i) no agreement, if K < 0; (ii) poor agreement, if 0 < K < 0.19; (iii) fair agreement, if 0.20 < K < 0.39; (iv) moderate agreement, if 0.40 < K < 0.59; (v) substantial agreement, if 0.60 < K < 0.79; and (vi) near perfect agreement, if 0.80 < K < 1.00 [24].

## 3. Results

### 3.1. Search and Selection of Publications

**Figure 1:**
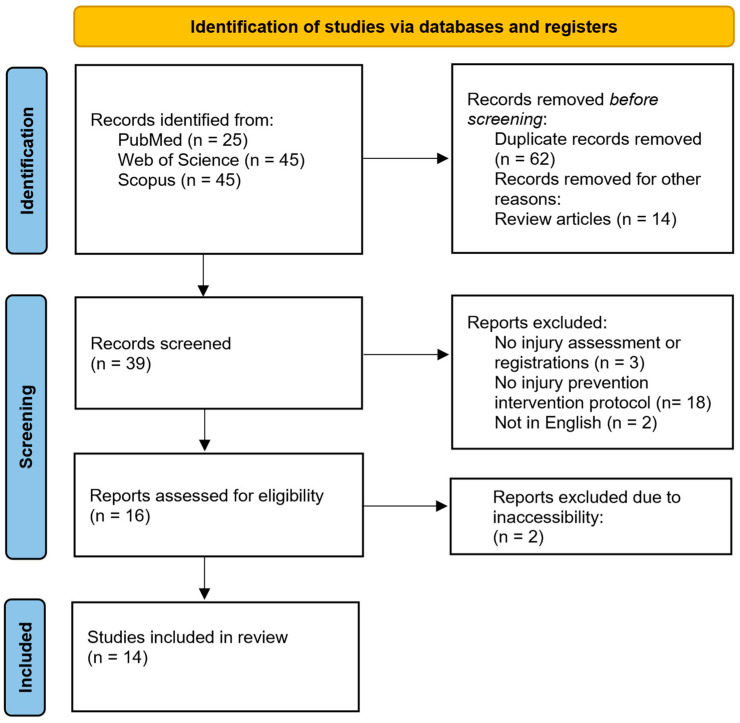
Flowchart of the systematic literature review.

The search of PubMed, Web of Science, and Scopus databases yielded 115 records. Sixty-two duplicates and fourteen review articles were removed. The remaining 39 full-text articles were read and assessed for eligibility, and 25 studies that did not meet the eligibility criteria were excluded. Thus, 14 articles [25,26,27,28,29,30,31,32,33,34,35,36,37,38] that met the criteria and objectives of this systematic review were included, as shown in Figure 1, which depicts the PRISMA flowchart for identifying, screening, and checking eligibility.

### 3.2. Quality and Risk of Bias of Individual Studies

The articles included in the final review stage had an average score of 17.29 ± 1.98 points (92.86%—rated as good quality). The main reason why some articles did not achieve a higher score was mainly due to the lack of information on the statistical power calculation. The average quality score of the 14 articles was 90.98 ± 10.41% (74–100%) based on the scoring using our modified Downs and Black Quality Assessment Checklist. The most common quality issues were failure to report subject characteristics, confounding variables, and true probability values (e.g., 0.035 instead of <0.05). Agreement between evaluators was almost perfect (K = 0.97, *p* < 0.001, 95% confidence intervals: 0.93–1.01).

### 3.3. Characteristics of the Included Studies

Table 1 presents key details from the studies included in the systematic review, focusing on the characteristics of each study, including the name of the first author, the program/protocol selected, its duration and frequency, the total sample, and the age group. The sample of all studies consisted of players ranging in age from U13 to Senior players. Interventions ranged in frequency from 1 to 3 times per week and in duration from 5 min to an entire season, depending on the program/protocol used. The main injury prevention practices included warm-up protocols, strength, neuromuscular, flexibility, stability-oriented, and Nordic hamstring exercises. Workload management was based on an RPE scale, before and after sessions, and other assessments using other equipment/methods were also applied.

### 3.4. Data Organization

For the purposes of this review, the studies were grouped into four broad areas according to the type of physical exercise used in the intervention program. These areas included (i) warm-up protocols (7 studies), (ii) proprioceptive training (3 studies), (iii) multicomponent programs (2 studies), and (iv) strength training (2 studies). The statistically significant main results and conclusions of the studies are presented in Table 2.

#### 3.4.1. Warm-Up Protocols

Several studies included in this review investigated the usefulness of warm-up protocols in preventing injuries in futsal players. FIFA 11+ is a well-known program that was investigated [30,31,32,37,38]. Research on the effects of FIFA 11+ on balance and proprioception produced mixed results. While some studies reported no significant change in static or dynamic balance or proprioception after 10 weeks of the program [30], others suggested long-term improvements in eccentric strength and balance [31]. However, several studies have shown that the FIFA 11+ program has a positive effect on injury reduction. Two studies found that players who performed the FIFA 11+ had a significantly lower risk of total, acute, and lower limb injuries over the course of the season compared to a control group [32,38]. In addition, the program can be a useful conditioning technique for improving the physical fitness and technical skills of young futsal players [37]. Futsal-specific warm-up routines are also promising. One study examined a multi-station exercise program as the final component of the warm-up. Compared to a control group, the effects of training load were reduced, but proprioceptive accuracy was increased [36]. This study also suggests that strict adherence to a systematic warm-up program may be essential to maximize injury reduction effects [36].

#### 3.4.2. Proprioceptive Training

Proprioceptive training may have a positive effect on injury prevention in futsal. One study found that landing mechanics improved after a specific proprioceptive training intervention [27]. The authors noted that improved awareness of joint position during the landing after jumping could potentially reduce the risk of lower extremity injury. Although not directly related to exercise interventions, studies of approaches to improve proprioception are also relevant. Low-dye taping is one such technique that has been shown to provide sensory feedback while also improving body awareness [29]. Although the study itself does not include training activities, the likely mechanism of improved proprioception warrants its inclusion in this area as it is consistent with the general purpose of this category. A study with a broader-ranging focus on injury prevention, such as workload management, also included a proprioceptive training component, albeit minimal. For example, a multicomponent program that included proprioceptive and neuromuscular training (only 2 and 3% of the total program duration, respectively) was examined and found to reduce overall injuries [35]. This suggests that even a small amount of proprioceptive training may be useful when combined with other injury-prevention interventions.

#### 3.4.3. Multicomponent Programs

Combining a variety of exercise strategies, such as multicomponent training programs, has been investigated as an option for injury prevention in futsal players. Some of the elements that are commonly included in such a program are strength training, flexibility exercises, balance training, and core stability work. A study using the Pilates method with two different protocols found that flexibility improved immediately after the intervention [25]. Although the flexibility gains were not significantly reduced after 15 days, it can be suggested that the Pilates method could be beneficial for improving the range of motion and potentially reducing the risk of injury. Another article compared a stability-focused intervention with a traditional strength program and again found significant improvements in core strength and trunk control in the stability group [28]. Increased core stability such as this may help players maintain appropriate posture and movement patterns during games, thereby reducing the risk of injury.

#### 3.4.4. Strength Training

(1) Strength training programs in general

One study looked at the effects of an HIIT program combined with NC exercise [26]. Although the authors did not find significant increases in isometric strength, this program resulted in significant improvements in intermittent work performance, a key aspect of futsal. Interestingly, while significant increases in isometric strength were not observed, there was a tendency for increased strength gains when longer training durations were applied or even different program designs. Thus, the authors suggested that strength training programs, such as HIIT-only or HIIT and Nordic hamstring exercises, could improve futsal performance and potentially reduce the risk of injury.

(2) Strength training based on isokinetic assessment

Although an isokinetic dynamometer assessment is not directly a strength training program, isokinetic assessments can be valuable tools for designing targeted programs. One study used an isokinetic dynamometer to assess the performance of the knee flexor (KF) and extensor (KE) muscles in futsal players. The study used a high-speed fatigue protocol [34]. The main findings suggested that this procedure resulted in a significant decrease in the performance of both the KF and KE muscles, with the KF showing the greatest decrease. In addition, the hamstring-to-quadriceps (H:Q) ratio, which measures muscle balance, decreased for all parameters except peak torque. The authors emphasized the importance of muscle balance when designing strength training programs. In this sense, isokinetic testing can provide useful information and help guide specific training programs to address this aspect, potentially reducing the risk of injury.

## 4. Discussion

The purpose of this study was to review the existing literature on injury prevention strategies for futsal players. The discussion was organized into the same four broad areas used in the Section 3: (i) warm-up protocols; (ii) proprioceptive training; (iii) multicomponent programs; and (iv) strength training.

### 4.1. Warm-Up Protocols

With regard to warm-up, the results provided strong evidence for the value of the FIFA 11+ program in reducing acute, total, and lower limb injuries in futsal players [32,37,38]. This program appears to be well adapted to the needs of futsal despite being primarily designed for soccer. Research has suggested that the program provides sustained improvements in eccentric strength and balance, which are critical for maintaining stability and control during intense movements on the hard court [31]. However, one study did not find significant improvements in static and dynamic balance or proprioception following a 10-week FIFA 11+ intervention [30]. However, another study reported significant improvements in quadriceps and hamstring strength, jumping performance, agility, and balance (fewer falls) following the program in youth futsal players [37]. These conflicting findings may be due to the differences in sample characteristics (e.g., age, experience level), fidelity of program implementation, or outcome measures used. On the other hand, one study examined a multi-station program and reported improvements in proprioceptive accuracy after the intervention and concluded that the effects of the program may persist after completion, although it may not sufficiently improve proprioceptive acuity and maximum vertical jump [36]. This suggests that such programs may provide additional benefits beyond those observed with the FIFA 11+. It is important to consider these potential limitations when evaluating the usefulness of multi-station protocols for reducing injury risk. Future research could compare the effects of different warm-up protocols, including multi-station programs alongside the FIFA 11+, to determine the most effective strategies for improving both physical performance and injury prevention in futsal players. Future studies should also investigate potential modifications to the FIFA 11+ program to maximize its usefulness for futsal players. For example, the addition of drills or exercises unique to futsal that replicate the rapid directional changes and close ball handling of the activity could increase its benefits in injury prevention. It would also be beneficial to investigate the long-term implementation and adherence rates of the FIFA 11+ program within futsal training regimes. In addition to structured warm-up routines, monitoring training load during training sessions may be another valuable alternative strategy for injury prevention. One study investigated the use of the RPE scale before and after training sessions and found that players with lower perceived exertion levels reported fewer injuries [33]. This suggests that coaches can use these tools, such as the RPE scale, alongside warm-up routines to manage training intensity and potentially reduce the risk of overtraining injuries. However, it is important to note that the RPE scale relies on subjective perception, and its results could be influenced by factors such as player honesty and fatigue levels. Future research could explore methods to improve the accuracy and objectivity of RPE-based training load monitoring. Understanding the feasibility and sustainability of these methods in real-world settings will facilitate the development of practical recommendations for coaches and teams.

### 4.2. Proprioceptive Training

Proprioceptive training strategies have been shown in the included studies to improve landing mechanics and possibly reduce the risk of lower extremity injuries, which are common in futsal [27]. By increasing body awareness and sensory feedback, methods such as low-dye taping may also be helpful, potentially leading to an improvement in joint stability during movement [29]. Notably, in multicomponent programs, even small amounts of proprioceptive training appeared to help when combined with other injury prevention strategies [35]. This finding suggests a beneficial effect when different approaches are implemented together. Future research could investigate the most appropriate volume and specific forms of proprioceptive training that work best for futsal players. It would also be beneficial to investigate how proprioceptive training affects injury rates and player performance over a longer period. To encourage widespread use in futsal programs, research into time and cost-effective proprioceptive training methods that could be incorporated into current training programs would also be helpful. Similar to football, ankle sprains are common in futsal due to the rapid changes in direction and the possibility of falling on a hard surface [39]. Studies in football have shown that proprioceptive training is effective in reducing the risk of ankle sprains and improving postural control [40,41,42]. Specific exercises used in football, such as wobble boards, single-leg balance training, or training with ankle disks, balance boards, and tilt boards, should be investigated and possibly modified for futsal injury prevention programs [43,44]. Proprioceptive training, which includes exercises that challenge balance and promote joint awareness, has the potential to prevent ankle sprains and improve movement control in futsal players, thereby reducing the risk of injury.

### 4.3. Multicomponent Training Programs

Research on multicomponent training programs that incorporate a variety of techniques, such as core stability exercises, flexibility training, balance training, and strength training, shows promise in reducing injuries in futsal players [25,28]. For example, the Pilates technique can increase the range of motion, which may reduce the risk of muscle sprains and tears [25]. Similar to stability training, it can improve trunk control and core strength, which can improve posture and potentially reduce the risk of injuries caused by imbalances or incorrect movement patterns [28]. Future research may investigate the ideal design and duration of multicomponent training programs for futsal players. In order to maximize their benefits, it would be beneficial to examine the specific elements of these programs that make the greatest contribution to injury prevention. Additionally, research into the long-term sustainability of these programs within training schedules and their impact on player performance would be informative. The usefulness of multicomponent programs to reduce overall injuries and muscular strain is often reported in the existing soccer literature [45], which may be applicable to futsal due to its similarities. As in football, futsal players can benefit from core strengthening exercises and programs that increase flexibility, as they improve core stability and movement control and reduce the risk of injury [46,47]. Core stability is essential for the effective transfer of force between the upper and lower body during basic futsal movements such as jumping, landing, and abrupt changes of direction [46]. For example, in the study by Owen et al. (2013) [45], a multicomponent program that included core stability, balance, and plyometric exercises performed twice a week for the duration of the season resulted in a significantly lower incidence of overall injuries in male professional football players compared to a control group. Given the similarities between the two sports, it is expected that futsal players will achieve similar results. Moreover, flexibility training may increase joint range of motion, facilitate more effective movement, and potentially reduce the incidence of muscle strain [48]. Muscle imbalances between the dominant and non-dominant leg may increase the risk of injury, according to football studies [49]. Futsal players can also benefit from multicomponent programs that include flexibility training to address these asymmetries and improve their overall movement mechanics.

### 4.4. Strength Training

Research suggests that Nordic hamstring exercises and high-intensity interval training (HIIT) are two strength training procedures that can improve intermittent work performance, a crucial component of futsal [26]. Improved performance often correlates with a reduced risk of injury, as athletes experience less fatigue and have better control of movement, while the direct influence on injury reduction requires further research. Furthermore, isokinetic evaluations may be useful tools in the development of focused strength training programs that target muscular imbalances that may be a factor in injuries [34]. Strength training programs can help athletes maintain ideal movement patterns and reduce their risk of injury by correcting imbalances. Future research could investigate the optimal intensity, volume, and frequency of strength training programs for futsal players, considering the specific demands of the sport and the potential for overtraining. It would also be beneficial to investigate how strength training affects injury rates, player performance, and the development of muscle strength and power over a longer period. Strength training is also commonly used in football, particularly in lower limb and core training, to prevent injuries such as muscle strains and ACL tears [45,48], which are also common in futsal [12]. Similar to football, futsal players may benefit from isokinetic training to identify and correct muscular imbalances, which may reduce their risk of injury [47]. Studies in football emphasize that addressing hamstring weakness or imbalances in relation to quadriceps strength is critical to preventing hamstring injuries [49,50]. This is also true for futsal players, and the included studies [26,31,34] took this into account. Further studies can investigate hamstring-specific strengthening routines that can be incorporated into futsal training programs with larger samples and for longer periods.

### 4.5. Key Findings and Considerations

This systematic review highlights the potential benefits of different injury prevention strategies for futsal players. Implementing a combination of the above approaches, including structured warm-up routines (shown to improve balance and reduce injury risk) [31,32,36,37,38], proprioceptive training (potentially improving landing mechanics and joint stability) [27], multicomponent programs (combining elements such as flexibility and core stability work to potentially reduce injury) [25,28], and strength training tailored to address muscle imbalances (which may improve performance and indirectly reduce injury risk) [26], appears to be promising. Overall, this review highlights the importance of a multi-pronged approach to injury prevention in futsal. Implementing a combination of these strategies appears to be a promising strategy for reducing injuries and promoting optimal player performance. By prioritizing injury prevention, coaches, trainers, and players can create a safer and more rewarding futsal experience for all involved.

### 4.6. Limitations and Future Directions

As with any systematic review, there are limitations. It is difficult to draw definitive conclusions about specific strategies because of the variability in study designs, interventions, and outcome measures. The primary objective of this review was to compile and synthesize existing evidence rather than to conduct primary research to determine causality. The different age ranges and competitive levels of participants across the studies also contribute to the complexity of drawing firm conclusions. Therefore, while the study provides valuable insights into different injury prevention strategies, the heterogeneity among studies requires caution in interpreting direct cause–effect relationships. Such efforts are essential to strengthen the overall body of evidence in futsal injury prevention. This highlights the need for future research with standardized protocols, larger sample sizes with a more diverse age range, and comparisons between different competitive levels to strengthen the overall body of evidence. It would also be beneficial to investigate the durability of injury prevention programs within training regimens and their long-term consequences. Research into futsal-specific injury processes would also be essential for the development of targeted prevention plans that take into account the inherent demands of the game, such as the smaller pitch, emphasis on rapid changes of direction, and the potential for collisions on a hard surface.

## 5. Conclusions

A multi-pronged approach combining warm-up routines, proprioceptive training, multicomponent programs, and strength training showed promise in reducing injuries in futsal players. However, the variability in study designs, interventions, and participant characteristics makes it difficult to draw definitive conclusions. Future research should prioritize high-quality studies with standardized protocols and larger sample sizes with a more diverse age range to provide more robust evidence. Investigating the long-term effectiveness and durability of these programs within training regimens is essential to developing sustainable injury prevention strategies. Furthermore, research into the long-term effects of these programs and futsal-specific injury strategies would be helpful in developing targeted prevention approaches.

## Figures and Tables

**Table 1:** Main characteristics of the studies included in the systematic review.

Study	Study Design and Program/Protocol	Duration	Frequency	Sample	Participant’s Age (Years)
Bertolla et al. [25]	Prospective, RCT Pilates method (2 protocols)	P1—familiarization (25 min, 2 weeks) P2—more advanced (25 min, 2 weeks)	3 times/week	11 collegiate players	17–20
Gómez et al. [26]	Prospective, RCT HIIT-only HIIT and NC	30–40 min, 4 weeks	1 time/week	21 federate players	18
Hamoongard et al. [27]	Prospective, RCT Neuromuscular with dual cognitive tasks	45–60 min, 8 weeks	3 times/week between 1 and 6 weeks 2 times/week during weeks 7–8	30 players	21.86 ± 3.27
Jebavy et al. [28]	Prospective, RCT Stability-oriented Traditional strength	30–40 min, 10 weeks	2 times/week: 1st; 4th; 5th; 8th; and 10th weeks 3 times/week: 2nd; 3rd; 6th; 7th; and 9th weeks	20 elite players	IG: 26 ± 8 CG: 27 ± 7
Klich et al. [29]	Prospective, Controlled Laboratory Study with a Repeated Measures Design LDT technique	72 h	Baseline, after 24 h and after 72 h	25 collegiate players	23.03 ± 1.15
Lopes et al. [30]	Prospective, RCT FIFA 11+	20 min, 10 weeks	2 times/week	61 amateur players	IG: 27.33 ± 4.33 CG: 25.55 ± 4.65
Lopes et al. [31]	Prospective, RCT FIFA 11+	20 min, 10 weeks + 10 weeks follow-up	2 times/week	58 amateur players	IG: 27.0 ± 5.1 CG: 26.0 ± 5.1
Lopes et al. [32]	Prospective, RCT FIFA 11+	20 min, 20 weeks	2 times/week	71 amateur players	IG: 27.0 ± 5.1 CG: 26.0 ± 5.1
Lorente et al. [33]	Retrospective, Observational, Longitudinal, Repeated-Measures RPE measured using the CR-10 Borg Scale	225 sessions, 40 weeks	Before and after each session	12 elite players	+18
Machado et al. [34]	Retrospective, Cross-sectional Isokinetic assessment	30 consecutive contractions at 300°/s	One-time session during pre-season	17 elite players	26.79 ± 6.45
Murillo et al. [35]	Retrospective, Longitudinal Reducing overall workload and intensity; RPE scale; proprioceptive training and neuromuscular control	Entire season	N/E	2016/2017: 12 elite players 2004/2005: 14 elite players	2016/2017: 27.00 ± 5.12 2004/2005: 29.00 ± 6.10
Pérez-Silvestre et al. [36]	Prospective, RCT Multi-station	10 min, 6 weeks	2 times/week	17 players	IG: 19.1 ± 2.3 CG: 19.0 ± 2.5
Reis et al. [37]	Prospective, RCT FIFA 11+	N/E	1.8 ± 0.1 times/week	36 adolescent players	17.3 ± 0.7
Tomsovsky et al. [38]	Prospective, RCT FIFA 11+	5 min	1 time/week	878 amateur teams	U13, U17, and senior

Data are presented as mean ± SD. Abbreviations: RCT = randomized control trial; CG = control group; IG = intervention group; P1 = protocol 1; P2 = protocol 2; HIIT = high-intensity interval training; NC = Nordic curl; N/E = nonexistent.

**Table 2:** Summary of the main results and conclusions of the included studies in the systematic review.

Study	Main Results	Conclusion
Bertolla et al. [25]	Fleximeter: significant differences (*p* < 0.01) were noted on the IG between the Pre (130.83° ± 13.63) and PI (140.17° ± 9.99) moments. Wells Bench: significant differences (*p* < 0.05) were noted on the IG between the Pre (36.50 ± 3.96 cm) and PI (38.83 ± 5.04 cm) moments.	↑ flexibility in the post-immediate with a non-significant decrease after 15 days. ↓ risk of injury due to triggered by the decrease in muscular length
Gómez et al. [26]	The HIIT + NC group and the HIIT group showed a significant improvement in intermittent work performance after the intervention (*p* = 0.04 and *p* = 0.01, respectively).	↑ intermittent work performance in both HIIT and HIIT + NC groups
Hamoongard et al. [27]	A significant improvement was noted in the IG compared to the CG for the dynamic knee valgus at IC (*p* = 0.02, ES = 0.31) and FF (*p* = 0.003, ES = 0.49), knee flexion at IC (*p* = 0.001, ES = 0.41) and FF (*p* = 0.001, ES = 0.32), ankle dorsiflexion at IC (*p* = 0.001, ES = 0.72) and FF (*p* = 0.002, ES = 0.50), and trunk flexion at FF (*p* = 0.001, ES = 0.59) angles.	↑ landing mechanics in players with knee ligament dominance defects
Jebavy et al. [28]	The IG had significantly improved the intraabdominal pressure test (*p* = 0.004), trunk flexion (*p* = 0.036), and side plank (*p* = 0.002) in posttest results.	↑ activation of functions of the DSS, and should be prioritized over traditional strength exercises in injury prevention training programs. The use of DSS might prevent injury and overloading in elite futsal players.
Klich et al. [29]	A decrease in FFD under the rearfoot (*p* ≤ 0.001) and forefoot (*p* ≤ 0.001) on the right and left sides. Increase in the plantar PPT in all regions of the foot (*p* ≤ 0.001).	Fascial taping can be an effective method for normalizing the FFD and reducing the PPT. The findings provide useful information regarding the prevention of and physical therapy for lower extremity injuries in soccer and futsal.
Lopes et al. [30]	The IG showed higher training exposure and lower BMI and BW.	Performing FIFA 11+ for 10 weeks did not improve static and dynamic balance as well as proprioception in amateur futsal players.
Lopes et al. [31]	In the long term, significant gains were obtained after adjustment for baseline differences in eccentric strength for both lower limbs as for the H:Q ratios for the dominant limb.	↑ long-term benefits in eccentric strength. ↑ long-term benefits in H/Q conventional and functional ratios of the knee of amateur futsal Players. ↓ injuries in amateur futsal players.
Lopes et al. [32]	Total injuries: 58 injuries during the futsal regular season (IG: 24; CG: 34). Incidence of injuries per 1000 player-hours: significantly higher in the control group (11.6 vs. 6.5). Acute injuries: significantly lower acute and lower limb injuries in the IG (11.2 vs. 5.7 and 8.7 vs. 4.4, respectively). Days injured: CG had a higher number of days injured (20.4 ± 17.3 vs. 10.5 ± 9.1).	↓ overall, acute, and lower limb injuries in amateur futsal players, during the season.
Lorente et al. [33]	The incidence of injuries was significantly lower (*p* < 0.05) among players with fewer warning signs (RPE of 6). In months with a higher training volume, warning signs were effective in reducing the number of injuries sustained by players.	↓ risk of injury when the coach was able to adjust training loads based on the players “warning signs”.
Machado et al. [34]	A significant (*p* < 0.01) time × muscle group interaction was observed. Significant reductions (*p* < 0.01) were noted in KF and KE performance for all parameters measured. KF showed a higher percentage decrease than KE. Significant reductions (*p* < 0.01) in the H:Q ratio were observed for work, average power, and peak power but not for peak torque.	The high-speed isokinetic fatigue protocol induced performance decrement in both KF and KE, with KF showing superior reductions. The H:Q ratio, calculated from work, average power, and peak power, decreased, contrasting with H:Q derived from peak torque. Peak torque measures exhibited less performance decrement compared to other assessments.
Murillo et al. [35]	In the 2016–2017 season, maintenance microcycles accounted for a higher percentage of injuries (53.6%), contrasting with ascending microcycles in 2004–2005 (58.3% *p* = 0.002). Injuries during training sessions decreased from 73.1% in 2004–2005 to 54.9% in 2016–2017 with a shift from overload to trauma as the primary cause (55.6% to 42.9%; *p* = 0.004). Despite the differences, the absolute number of injuries decreased in the 2016–2017 season.	The measures adopted were effective in achieving a significant reduction in the incidence of injuries in the 2016–2017 season compared to the 2004–2005 season.
Pérez-Silvestre et al. [36]	Significant group-by-time interactions in AAE, with CG presenting higher values at Post10wk compared to baseline, while the experimental group exhibited a reduction at Post6wk and Post10wk (*p* = 0.028). CG had higher values of AAE than the experimental group at Post10wk (*p* = 0.050, d = 0.8). The main time effect in RAE with the control group showing higher values at Post10wk compared to baseline (*p* = 0.004, d = 0.7). IG exhibited lower values of VAE compared to the control group at Post10wk (*p* = 0.039, d = 1.2).	↑ proprioceptive precision. The effects of the program may persist after it ends, although it may not sufficiently improve proprioceptive acuity and maximum vertical jump. ↑ precision and ↓ training load effects.
Reis et al. [37]	IG increased (*p* < 0.05) quadriceps concentric (14.7–27.3%) and hamstrings concentric (9.3–13.3%) and eccentric (12.7%) peak torque. IG improved the functional H:Q ratio by 1.8% to 8.5% (*p* < 0.05). IG improved (*p* < 0.05) SJ (13.8%) and CMJ (9.9%) and 5-m and 30-m sprint (8.9% and 3.3%, respectively), agility (4.7%), and slalom (4.8%) performances. IG also improved balance by decreasing the number of falls by 30% in the nondominant limb.	The FIFA 11+ can be used as an effective conditioning means for improving physical fitness and technical performance of youth futsal players, potentially enhancing performance, technical skills, and reducing injury risk when completed as a warm-up routine.
Tomsovsky et al. [38]	IG showed a significantly lower rate of contact injuries (RR ¼ 0.68, 95% CI ¼ 0.51 to 0.98). Subgroup analysis, based on the warm-up adherence of intervention teams (low, intermediate, high), showed a lower rate of all injuries (RR ¼ 0.52, 95% CI ¼ 0.29 to 0.97), and LE injuries (RR ¼ 0.32, 95% CI ¼ 0.14 to 0.81) in the high compared to low adherence group.	A futsal-specific warm-up can lower the incidence of contact injuries in amateur players. With high adherence, the occurrence of all injuries, including LE injuries, may decrease.

Data are presented as mean ± SD. Abbreviations: ↑ = increased; ↓ = decreased; CG = control group; IG = intervention group; PI = Post-Immediate; IC = initial contact; FF = full flexion; DSS = Deep stabilization system; LDT = low-dye taping; FFD = foot force distribution; PPT = plantar pressure threshold; BMI = body mass index; BW = body weight; RPE = rated perceived exertion; H:Q ratio = hamstring to quadricep ratio; KF = knee flexion; KE = knee extension; AAE = absolute angle error; RAE = relative angular error; LE = low extremity.

## Data Availability

No new data were created or analyzed in this study.
